# Prognostic factors in patients with T1 glottic cancer treated with radiotherapy

**DOI:** 10.1007/s00066-019-01481-2

**Published:** 2019-06-18

**Authors:** A. Mucha-Małecka, A. Chrostowska, K. Urbanek, K. Małecki

**Affiliations:** 1 Clinic of Oncology and Department of Radiotherapy, Maria Skłodowska-Curie Memorial Cancer Center and Institute of Oncology, Cracow Branch, Garncarska 11, 31-115 Cracow, Poland; 2 Department of Radiotherapy for Children and Adults, University Children’s Hospital of Cracow, Wielicka 265, 30-663 Cracow, Poland

**Keywords:** Laryngeal neoplasms, Glottis, Malnutrition, Commissure infiltration, Treatment outcome, Larynxtumoren, Glottis, Malnutrition, Infiltration der Kommissur, Behandlungsergebnis

## Abstract

**Purpose:**

Presentation of long-term results of radiation treatment in patients with T1 glottic cancer and evaluation of prognostic factors.

**Methods:**

We performed a retrospective analysis in a group of 569 patients with T1 squamous cell glottic carcinoma treated with radiotherapy at the Center of Oncology in Cracow between 1977 and 2007. In all, 503 (88%) patients presented with T1a stage disease and 66 (12%) with T1b. Anterior commissure infiltration was present in 179 (31%) patients. Average hemoglobin level prior to therapy was 13.9 g/dl. Using the body mass index (BMI), 114 (20%) patients were underweight, and 91 (16%) were overweight. Median time between collecting tumor specimen and beginning of radiotherapy was 56 days (range 14–145 days). Treatment regimen was normofractionated with single fraction ≤2 Gy in 102 (18%) and hypofractionated in 467 (82%) patients.

**Results:**

The 5‑ and 10-year overall survival (OS), disease-specific survival (DSS) and local control (LC) rates were 85 and 68%, 88 and 86%, 89 and 87%, respectively. Multivariate analysis showed that tobacco smoking, low hemoglobin level (<13 g/dl), anterior commissure infiltration, fraction dose ≤2 Gy and time from collecting specimen to beginning of therapy longer than 30 days had negative impact on LC and DSS. Patients’ age over 60 years, worse performance status and malnutrition (BMI <18.5) had negative impacts on OS.

**Conclusions:**

Radiotherapy is a highly effective treatment method in patients with T1N0M0 glottic cancer. LC and DSS may be improved following hypofractionation, smoking cessation, and shortening of waiting-time until start of treatment. OS was mainly influenced by nutritional and performance status.

## Zusammenfassung

**Ziel:**

Darstellung der langfristigen Ergebnisse einer Strahlentherapie bei Patienten mit T1-Glottiskarzinom und Beurteilung der prognostischen Faktoren.

**Methoden:**

In die retrospektive Analyse wurden 569 Patienten mit T1-Plattenepithelkarzinom der Glottis eingeschlossen, bei denen zwischen 1977 und 2007 im Tumorzentrum Krakau eine Strahlentherapie durchgeführt wurde. Bei 503 Patienten (88%) wurde der Status T1a und bei 66 (12%) der Status T1b diagnostiziert. Eine Infiltration der Commissura anterior wurde bei 179 Patienten (31%) festgestellt. Der Ausgangshämoglobinwert betrug durchschnittlich 13,9 g/dl. In Bezug auf den Body-Mass-Index (BMI) wurde bei 114 Patienten (20%) Untergewicht beobachtet und bei 91 (16%) Übergewicht. Die mittlere Zeit zwischen der Probenentnahme und dem Beginn der Strahlentherapie betrug 56 Tage (Bereich 14–145 Tage). Die Strahlentherapie war normofraktioniert mit einer Fraktionsdosis ≤2 Gy bei 102 Patienten (18%) und hypofraktioniert bei 467 Patienten (82%).

**Ergebnisse:**

Das Gesamtüberleben (OS), das krankheitsspezifische Überleben (DSS) und die Rate der lokalen Kontrolle (LC) nach 5 und 10 Jahren betrugen 85 und 68%, 88 und 86% sowie 89 und 87%. Eine multivariate Analyse bewies, dass Nikotinabusus, ein niedriger Hämoglobinwert (≤13 g/dl), eine Infiltration der Commissura anterior, eine Fraktionsdosis ≤2 Gy und eine Zeitspanne zwischen der Probenentnahme und dem Beginn der Strahlentherapie von mehr als 30 Tagen die LC und das DSS negativ beeinflussten. Einen ungünstigen Einfluss auf das OS hatten ein Patientenalter über 60 Jahre, ein schlechter Performance-Status und Unterernährung (BMI <18,5 kg/m^2^).

**Schlussfolgerungen:**

Die Strahlentherapie ist eine hochwirksame Behandlungsmethode für Patienten mit T1N0M0-Glottiskarzinom. LC und DSS können nach Hypofraktionierung, Raucherentwöhnung und Verkürzung der Wartezeit bis zum Beginn der Strahlentherapie verbessert werden. Das OS wurde hauptsächlich durch den Ernährungs- und Performance-Status beeinflusst.

## Introduction

Laryngeal cancer is the most common malignancy of the head and neck region and constitutes 2–4% of all malignant neoplasms. In Poland in 2015, 2536 new cases and 1610 deaths caused by laryngeal cancer were reported [1]. The most common location of this cancer is the glottis [2]. Thus, hoarseness—as a cancer-related symptom—facilitates diagnosis at early stages [3], leading to good treatment results [4–10]. There are two equally efficient treatment methods for patients with T1 laryngeal cancer—radiotherapy and surgery (performed transorally with a laser). There are limited prospective data showing a clear advantage of any of these methods. Treatment results come mostly from retrospective trials and are comparable. The 5‑year local control for radiotherapy ranges from 80–95%, and from 82–100% for transoral laser surgery [4, 8–15]. The treatment method is selected based mainly on institutional experience and the patient’s preferences. In most institutions, radiotherapy is preferred due to better voice quality after treatment [16, 17]. The basic goal of therapy is to give a patient the highest chance of cure, as well as minimize the risk of side effects and ensure the best possible functional result.

The aim of the study was to evaluate long-term results following radiotherapy in a large cohort of patients with T1 laryngeal cancer and to analyze the prognostic factors affecting treatment outcome.

## Materials and methods

Basing on data included in the medical records we performed a retrospective analysis of a group of 569 patients with T1N0M0 laryngeal cancer, treated at the Center of Oncology in Cracow between 1977 and 2007. In Table 1, data concerning all analyzed patients are summarized.

**Table 1:** Patient characteristics

Parameter	Category	Number of patients *n* (%)
Sex	Male	542 (95)
	Female	27 (5)
Age	≤60 years	277 (49)
	>60 years	292 (51)
Performance status (ZUBROD)	0–1	502 (88)
	2–3	67 (12)
Tumor stage (T)	T1a	503 (88)
	T1b	66 (12)
Anterior commissure infiltration	No	390 (69)
	Yes	179 (31)
Tumor grading (WHO)	G1	297 (52)
	G2	176 (31)
	G3	12 (2)
	GX	84 (15)
Hemoglobin level	≥13 g/dl	465 (82)
	<13 g/dl	104 (18)
BMI (body mass index; kg/m^2^)	<18.5	114 (20)
	≥18.5 < 25	364 (64)
	≥25	91 (16)
Cigarettes smoked per day	0	80 (14)
	≤20	48 (8)
	>20	441 (78)
Pack–years	0	80 (14)
	<40	210 (37)
	≥40	279 (49)
Alcohol use	No	407 (72)
	Yes	162 (28)
Time interval biopsy–treatment	≤30 days	148 (26)
	>30 days	421 (74)
Radiotherapy technique	1–2 oblique wedged beams	397 (69.7)
	2–2 opposite wedged beams	170 (30)
	3–1 mixed photon–electron beam	2 (0.3)
Total dose	≤60 Gy	467 (82)
	>60 Gy	102 (18)
Fraction size	Normofractionated (≤2 Gy)	102 (18)
	Hypofractionated (>2 Gy)	467 (82)
Overall treatment time (OTT)	≤36 days	363 (64)
	>36 days	206 (36)

*GX* no data on differentiation

There were 542 males (95%) and 25 females (5%) in the analyzed group. Mean age was 60 years (range 27–87 years), 502 (88%) patients had very good and good performance status (ZUBROD 0–1), 62 patients (11.5%) had ZUBROD 2, and only 3 patients’ (0.5%) performance status was ZUBROD 3. Tumor location and stage were determined based upon indirect laryngoscopy and radiology—CT (computed tomography) or MRI (magnetic resonance imaging) scan.

There were 503 (88%) patients with T1a cancer, and the remaining 66 (12%) with T1b. Anterior commissure infiltration was present in 179 patients (31%). All of the patients had squamous cell carcinoma, including well-differentiated (G1) in 297 (52%) patients, moderately differentiated (G2) in 176 (32%) patients, and poorly differentiated in 12 patients (2%). In 84 patients (15%), the degree of differentiation was not assessed. The median hemoglobin level prior to treatment was 13.2 g/dl (interquartile range 9.8–13.9 g/dl). Based upon the data from medical history, the nutrition level was assessed. The patients were divided into subgroups, depending on body mass index (BMI): <18.5—underweight (114 patients—20%); 18.5–25—normal nutrition (364 patients—64%); >25—overweight (91 patients—16%). In the analyzed group, only 80 (14%) patients did not have a history of smoking, the remaining smoked between 2 and 60 cigarettes per day (median 20, interquartile range 15–20). The median smoking time was 37 years (interquartile range 28–46). The average patient smoked 28 pack–years (median 26, interquartile range 10–42) and 162 patients (28%) were heavy alcohol drinkers. Heavy drinking was defined as consuming 15 drinks or more per week for men, and 8 drinks or more per week for women.

In the analyzed group, three radiotherapy techniques were used. In 397 patients (69.7%), two oblique wedged beams and in 170 patients (30%) two opposite wedged beams of 60 cobalt or 6 MV linac photons were used. In 2 patients (0.2%), single mixed photon–electron beam was used. The last technique was used in patients who suffered from comorbidities such as neurological or degenerative spine disorders, because of which they could not be treated in therapeutic position with head bent backwards. The CTV (clinical target volume) included entire larynx with false and true vocal cords, anterior and posterior commissures, arytenoids and aryepiglottic folds and subglottic space. An additional margin of 5 mm was given around CTV to make the PTV (planning target volume). Finally PTV extended from the top of the thyroid cartilage to the bottom of the cricoid cartilage with an anterior skin fall-off. In 21 (12%) patients with anterior commissure infiltration treated after 2000, an anterior flab was used. All the patients were irradiated once a day, 5 times a week, to a total dose of 60–70 Gy (average dose 61 Gy), with fraction doses of 2–2.5 Gy. Mean treatment time was 36 days (range 32–58 days). The mean waiting time to start of treatment, measured from the collection of tumor specimen for histopathology to the first day of radiotherapy, was 56 days (range 14–145 days).

The analysis was carried out retrospectively based on data included in the medical records of patients treated over 30 years from 1977 to 2007. All patients gave informed consent for the treatment and scientific analysis of medical records. No additional approval of the bioethical committee was obtained because the applied treatment was not experimental.

The 5‑ and 10-year overall survival and local control rates were calculated using Kaplan–Meier method. To analyze differences between groups, the log-rank and chi-square (χ^2^) tests were performed, with statistically significant *p* value of <0.05. Independent prognostic factors were selected using multivariate Cox proportional hazards regression model. For Cox multivariate analysis, the variables statistically significant in the univariate analysis were included.

## Results

In the analyzed group, the mean observation period was 118 months. During this time, 185 patients (33%) died, whereby 152 (27%) died of non-oncological causes. In the remaining group of 33 patients, laryngeal cancer was the cause of death in 21 (4%) cases, and 12 patients (2%) died because of second malignancy—lung cancer. Overall treatment tolerance was good—in 549 (96%) patients radiotherapy was completed according to the treatment plan. Acute and late toxicities were evaluated using the RTOG (Radiation Therapy Oncology Group) scale. Grade 0 (G0) acute mucosal toxicity was observed in 31 (6%) patients, GI in 224 (39%) patients, GII in 275 (48%) patients, and GIII in 39 (7%) patients. No acute skin toxicity (G0) was reported in 434 (76%) patients, 122 patients (21%) developed GI skin toxicity, 9 (2%) patients had GII, and 4 (1%) patients had GIII skin toxicity. Late toxicity was reported in 51 (9%) patients. The most frequent side effects were arytenoid edema observed in 27 (5%) patients, vocal cord fibrosis in 25 (4%) patients, chronic hoarseness in 25 (4%), and xerostomia in 9 patients (1.5%).

**Fig. 1:**
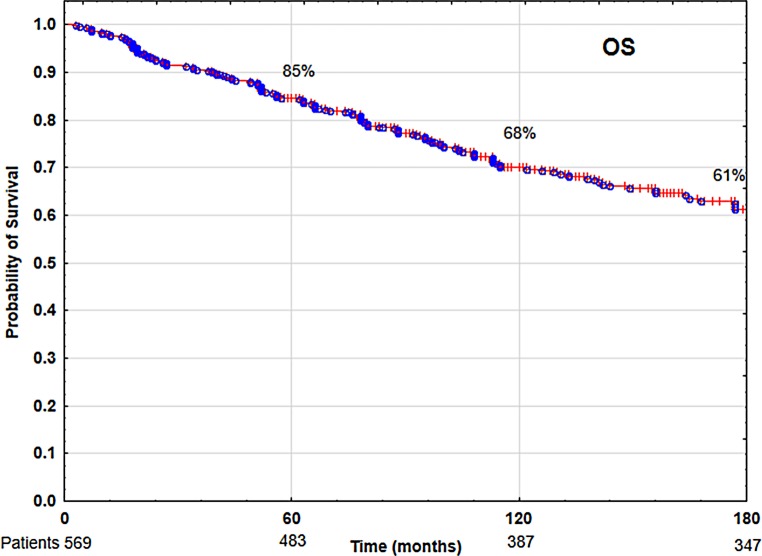
Overall survival (OS) rate

**Fig. 2:**
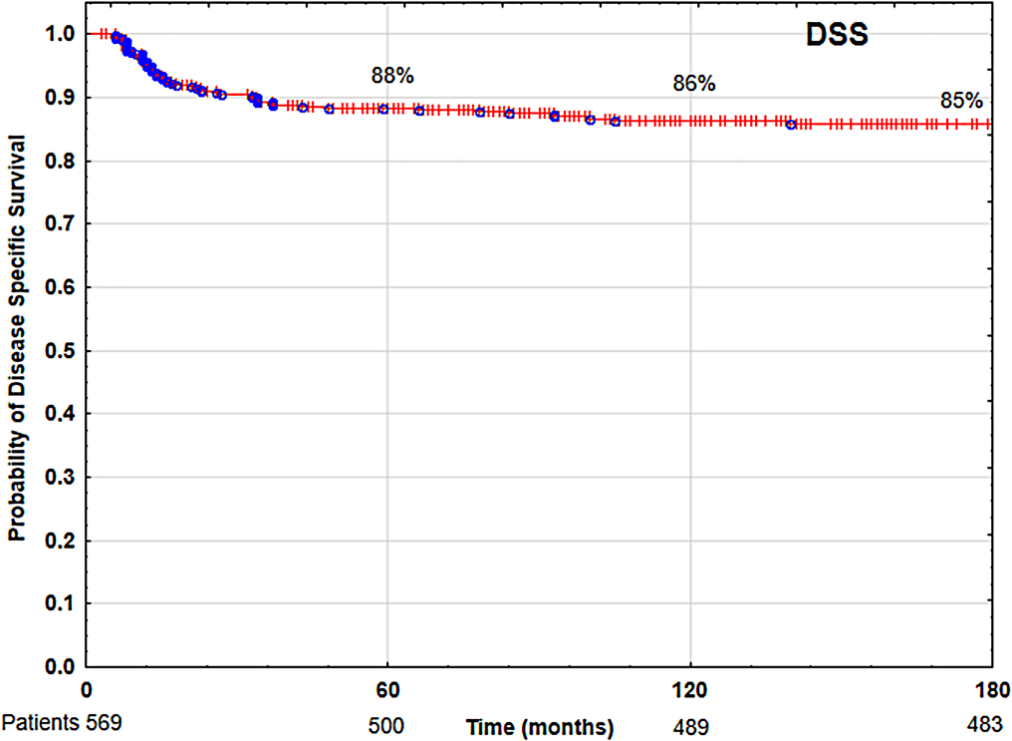
Disease-specific survival (DSS) rate

**Fig. 3:**
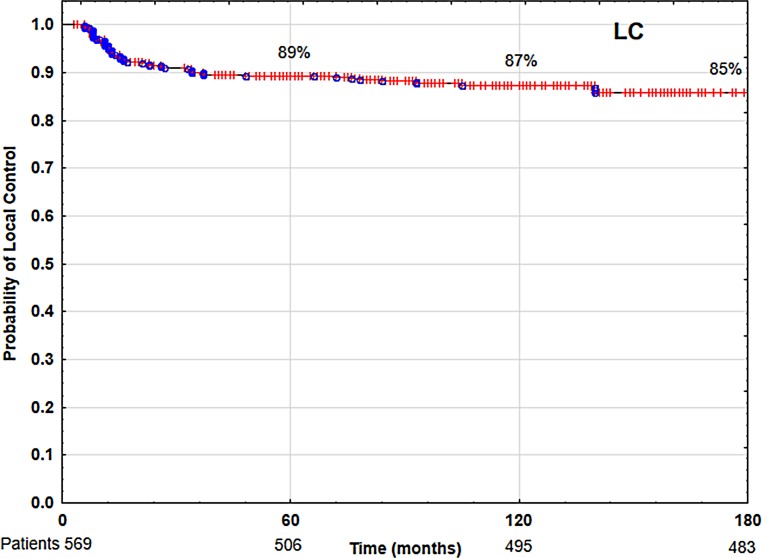
Local control (LC) rate

In the analyzed group of 569 patients with T1N0M0 laryngeal cancer, the 5‑ and 10-year overall survival (OS) rates, disease-specific survival (DSS) rates, and local control (LC) rates were 85 and 68% (Fig. 1), 88 and 86% (Fig. 2), 89 and 87% (Fig. 3), respectively. Local recurrence developed in 72 patients (13%). Most of them occurred within the first two years after radiotherapy (48 cases, 67%); the remaining 14 patients developed local relapse during the next 3 years. Median time to relapse was 14 months. Salvage surgical therapy was performed in 53 (74%) patients, whereby total laryngectomy was necessary in 16 cases due to the extent of the relapse. One patient had salvage radiotherapy. In 18 patients (25%) salvage therapy was impossible to perform due to locally advanced disease, poor performance status or lack of patient’s consent. The 5‑ and 10-year local control rates, including patients treated for local relapses, were 94 and 90%, respectively. In the 5‑year observation period, larynx preservation was achieved in 92% of patients, in the 10-year period in 88% of patients.

The results of univariate analysis of correlations between selected clinical features and 5‑ and 10-year local control, overall survival and disease-specific survival rates are shown in Table 2, while the results of univariate analysis of correlations between selected aspects of radiotherapy and 5‑ and 10-year local control, overall survival and disease-specific survival rates are shown in Table 3.

**Table 2:** Results of univariate analysis of correlations between selected clinical features and 5‑ and 10-year local control (LC), overall survival (OS), and disease-specific survival (DSS) rates

Parameter	Category	5-year LC	10-year LC	5-year DSS	10-year DSS	5-year OS	10-year OS
Sex	Male	89%	87%	88%	86%	84%	70%
	Female	89%	89%	89%	89%	84%	71%
		NS	NS	NS	NS	NS	NS
Age	≤60 years	85%	82%	84%	82%	91%	81%
	>60 years	93%	93%	91%	90%	75%	58%
		*p* = 0.001	*p* = 0.001	*p* = 0.010	*p* = 0.010	*p* < 0.001	*p* < 0.001
Performance status (ZUBROD)	0–1	88%	87%	88%	86%	88%	71%
	2–3	94%	90%	92%	88%	70%	52%
		NS	NS	NS	NS	*p* = 0.004	*p* = 0.004
Tumor stage (T)	T1a	90%	88%	88%	86%	90%	80%
	T1b	82%	82%	86%	83%	82%	69%
		NS	NS	NS	NS	*p* = 0.012	*p* = 0.012
Anterior commissure infiltration	No	92%	90%	90%	88%	85%	72%
	Yes	77%	70%	75%	73%	79%	69%
		*p* = 0.005	*p* = 0.005	*p* = 0.004	*p* = 0.004	NS	NS
Tumor grading (WHO)	G1	91%	89%	90%	89%	86%	72%
	G2	90%	89%	87%	84%	82%	70%
	G3	90%	90%	91%	91%	92%	81%
		NS	NS	NS	NS	NS	NS
Hemoglobin level	≥13 g/dl	92%	91%	91%	90%	84%	71%
	<13 g/dl	75%	70%	75%	71%	75%	70%
		*p* < 0.001	*p* < 0.001	*p* < 0.001	*p* < 0.001	NS	NS
BMI (Body Mass Index) (kg/m^2^)	<18.5	73%	69%	73%	70%	72%	60%
	≥18.5 < 25	92%	91%	92%	90%	86%	69%
	≥25	95%	94%	94%	92%	89%	85%
		*p* < 0.001	*p* < 0.001	*p* < 0.001	*p* < 0.001	*p* = 0.003	*p* = 0.003
Cigarettes smoked per day	0	97%	95%	96%	96%	88%	78%
	≤20	90%	89%	88%	87%	80%	70%
	>20	81%	79%	81%	78%	80%	58%
		*p* = 0.007	*p* = 0.007	*p* = 0.004	*p* = 0.004	NS	NS
Pack–years	0	97%	95%	96%	96%	88%	78%
	<40	90%	90%	89%	87%	82%	78%
	≥40	84%	82%	83%	82%	82%	58%
		*p* = 0.021	*p* = 0.021	*p* = 0.008	*p* = 0.008	*p* = 0.004	*p* = 0.004
Alcohol use	No	90%	89%	89%	87%	88%	72%
	Yes	88%	87%	86%	85%	81%	67%
		NS	NS	NS	NS	*p* = 0.022	*p* = 0.022
Time interval biopsy–treatment	≤30 days	90%	89%	90%	89%	89%	70%
	>30 days	85%	83%	83%	79%	80%	67%
		NS	NS	*p* = 0.006	*p* = 0.006	NS	NS

*NS* no significance

**Table 3:** Results of univariate analysis of correlations between selected aspects of radiotherapy and 5‑ and 10-year local control (LC), overall survival (OS) and disease-specific survival (DSS) rates

Parameter	Category	5-year LC	10-year LC	5-year DSS	10-year DSS	5-year OS	10-year OS
Radiotherapy technique	1	92%	89%	91%	90%	80%	60%
	2	88%	87%	86%	85%	84%	74%
	3	100%	100%	100%	100%	100%	0%
		NS	NS	NS	NS	*p* = 0.016	*p* = 0.016
Total dose	≤60 Gy	91%	90%	89%	87%	84%	74%
	>60 Gy	81%	78%	82%	78%	76%	50%
		*p* = 0.002	*p* = 0.002	*p* = 0.012	*p* = 0.012	*p* < 0.001	*p* < 0.001
Fraction size	≤2 Gy	79%	70%	77%	70%	70%	48%
	>2 Gy	90%	90%	90%	90%	85%	75%
		*p* < 0.001	*p* < 0.001	*p* < 0.001	*p* < 0.001	*p* < 0.001	*p* < 0.001
Overall treatment time	≤36 days	92%	91%	90%	89%	88%	80%
	>36 days	84%	80%	84%	80%	72%	53%
		*p* = 0.001	*p* = 0.001	*p* = 0.005	*p* = 0.005	*p* < 0.001	*p* < 0.001

**Fig. 4:**
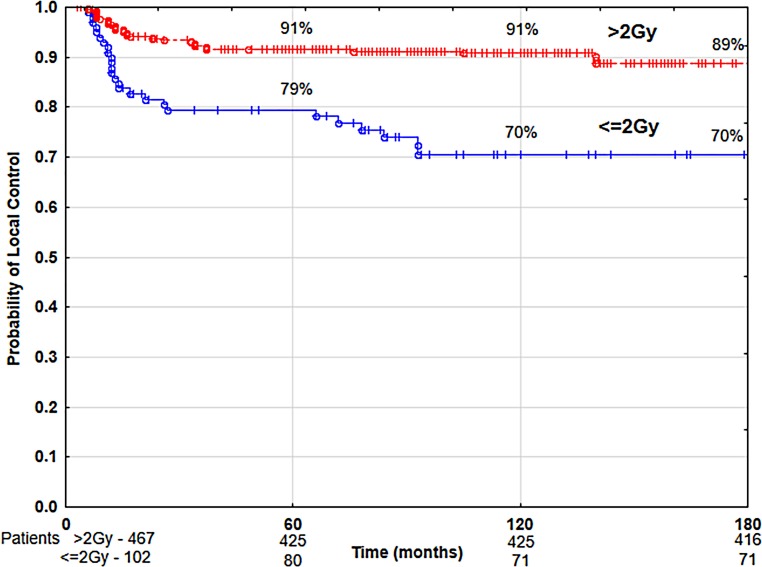
Local control rate according to fractionation

**Fig. 5:**
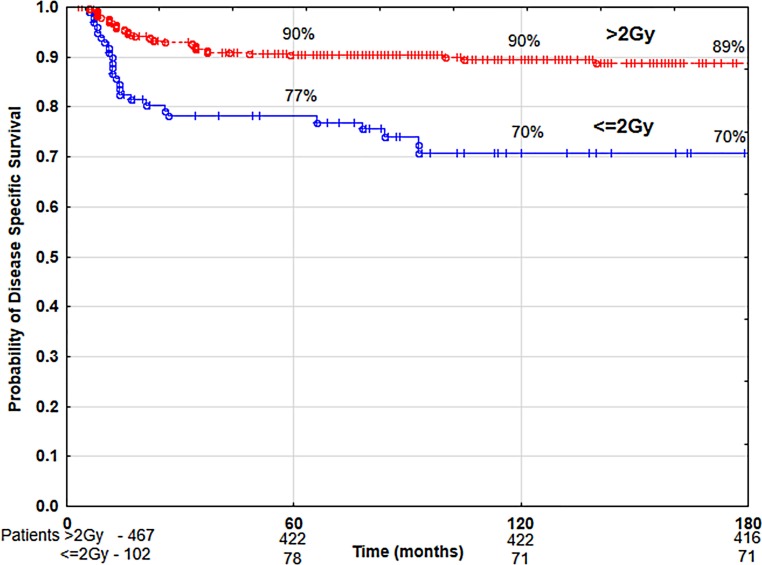
Disease-specific survival rate according to fractionation

**Fig. 6:**
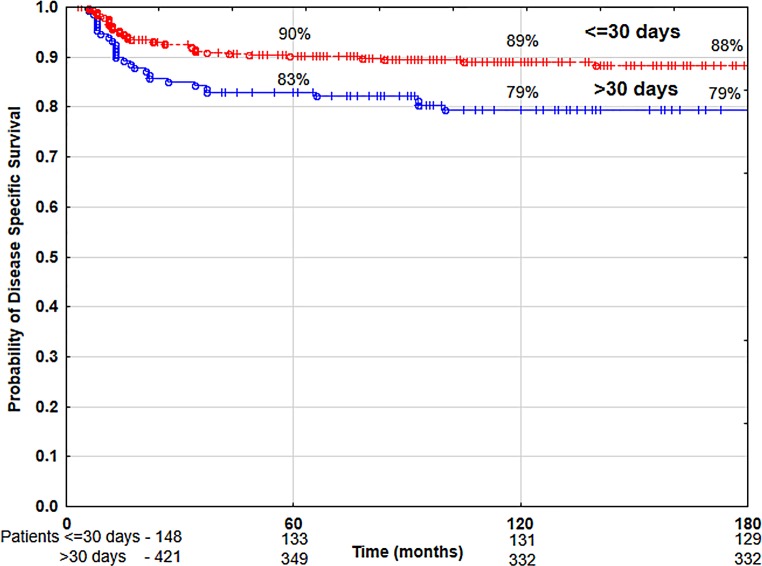
Disease-specific survival rate according to treatment delay

The univariate analysis showed that the following had a statistically significant negative impact on local control and disease-specific survival rates: patients’ age below 60 years, anterior commissure infiltration, hemoglobin level below 13 g/dl, malnutrition (BMI level below 18.5 kg/m^2^), tobacco smoking (particularly more than 20 cigarettes per day over a period of time—more than 40 pack–years) (Table 2), application of normofractionated radiotherapy (≤2 Gy), total dose higher than 60 Gy, and overall treatment time longer than 36 days (Table 3). Time from obtaining tumor sample to beginning of radiotherapy had statistically significant negative impact on disease-specific survival rates if it was longer than 30 days (Table 2). The following had a negative impact on overall survival: patients’ age over 60 years, poorer (ZUBROD 2–3) performance status, more advanced tumor stage (T1b), malnutrition (BMI <18.5), high lifetime tobacco exposure (>40 pack–years), alcohol abuse (Table 2) and normofractionated radiotherapy (≤2 Gy), total dose over 60 Gy, and treatment time longer than 36 days (Table 3). The use of hypofractionated radiotherapy (>2 Gy) was associated with the improvement of 10-year LC and DSS by 20% and OS by 22% compared to the group of patients treated with normofractionated radiotherapy (≤2 Gy) (Figs. 4 and 5). The recurrence risk in the group of patients treated with normofractionated radiotherapy (≤2 Gy) was 3 times higher than in the group treated with hypofractionated radiotherapy (>2 Gy) (Table 4), similarly the risk of death from cancer was 3.1 times higher (Table 5). In patients who started radiotherapy up to 30 days after the collection of tumor specimen for histopathology the 5‑year and 10-year DSS was 90 and 89%, respectively, compared to 83 and 79% for patients with waiting times above 30 days (Fig. 6). The multivariate analysis showed that an increase in the waiting time to start of radiotherapy beyond 30 days was associated with a 1.7-fold increased risk of death from cancer (Table 5).

**Table 4:** Results of multivariate analysis of relationship between selected prognostic factors and local control

Parameter	Category	*N*	Relative risk	*p* value
Cigarettes smoked per day	0	80	1.0	<0.001
	≤20	48	1.8	
	>20	441	3.3	
Anterior commissure infiltration	No	390	1.0	0.002
	Yes	179	3.2	
Hemoglobin level	≥13 g/dl	465	1.0	0.003
	<13 g/dl	104	2.9	
Fraction size	>2 Gy	467	1.0	<0.001
	≤2 Gy	102	3.0

The multivariate analysis showed that tobacco smoking, low hemoglobin level and fraction dose had statistically significant impact on local recurrence risk and risk of treatment failure (Tables 4 and 5). Risk of treatment failure was also affected by time from collecting tumor sample to beginning of radiotherapy. Infiltration of anterior commissure had an impact on local recurrence risk (Table 4). The multivariate analysis also revealed that patients’ age, performance and nutritional status prior to treatment had a significant impact on the risk of death (Table 6).

**Table 5:** Results of multivariate analysis of relationship between selected prognostic factors and risk of disease-specific survival

Parameter	Category	*N*	Relative risk	*p* value
Fraction size	>2 Gy	467	1.0	<0.001
	≤2 Gy	102	3.1	
Cigarettes smoked per day	0	80	1.0	<0.001
	≤20	48	1.6	
	>20	441	3.2	
Hemoglobin level	≥13 g/dl	465	1.0	<0.001
	<13 g/dl	104	2.7	
Time interval biopsy–treatment	≤30 days	148	1.0	0.021
	>30 days	421	1.7

**Table 6:** Results of multivariate analysis of relationship between selected prognostic factors and overall survival

Parameter	Category	*N*	Relative risk	*p* value
Age	≤60 years	277	1.0	<0.001
	>60 years	292	2.6	
Performance status (ZUBROD)	0–1	502	1.0	0.015
	2–3	67	1.5	
Body mass index (kg/m^2^)	≥18.5	455	1.0	0.030
	<18.5	114	1.9	

## Discussion

We present data on a total of 569 patients with early glottic carcinoma following radical radiation treatment. Results are well in line with data from the literature and demonstrate excellent local control rates of 89 and 87% at 5 and 10 years, respectively.

Numerous literature data show local control rates of 79–93% and disease-specific survival rates of 91–92% after radiotherapy compared to 73–89% and 91–98%, respectively, after surgery [5–7, 11, 13, 14, 18, 19]. The review of 36 publications performed by O’Hara et al. showed no difference in local control between radiotherapy and laser surgery: 3‑year local control rates for T1a tumors were 89.3% for radiotherapy and 88.9% for laser surgery, and in T1b tumors 86.2 and 76.8%, respectively [14]. Some authors suggest that there is not enough evidence to consider radiotherapy or surgery superior in treatment of T1 laryngeal cancer [9]. Others report significantly better results of radiotherapy compared to chordectomy [18, 19]. In recent years, the use of open surgery is becoming less frequent, due to higher voice quality after radiotherapy and endolaryngeal surgery [12].

The presented treatment results show high efficacy of both radiotherapy and laser surgery; thus, voice quality after therapy may affect the choice between them. In our study, the occurrence of hoarseness after 6 months and later from the end of radiotherapy (as a late radiation-induced reaction) was reported in 25 patients (4%). The results of studies concerning this topic are vague. Remmelts et al. compared voice quality in patients who underwent radiotherapy or laser surgery. The results of subjective analysis of voice quality were similar in both groups for T1a tumors, and for T1b tumors voice quality was worse after laser surgery. The authors conclude that in more advanced tumors laser surgery must be deeper, thus, negatively impacting voice quality [20]. In a systematic review and meta-analysis of 19 randomized trials, Abdurehim et al. showed no difference in voice quality of patients with T1a laryngeal cancer treated with radiotherapy or surgery [10]. Aaltonen et al. in a prospective trial analyzed voice quality in patients with T1a larynx cancer, treated with laser surgery or radiotherapy. In general, voice quality assessed by the authors was similar for both groups; however, 2 years after completing treatment, the frequency of hoarseness worsening the quality of life was higher in the group treated with a laser [16]. Higgins et al. performed a meta-analysis of 7600 patients with early glottis cancer and reported better voice quality after radiotherapy versus transoral laser surgery [17]. High efficacy and plausible voice quality after radiotherapy justify frequent application of this treatment option in patients with T1 glottis cancer.

Overall treatment tolerance was good in our group of patients. Surprisingly no acute skin toxicity was reported in 434 (76%) patients. This observation may be a result of underestimation of GI acute skin reactions in medical records. In the literature radiation dermatitis is common side effect developing in the majority of patients receiving radiotherapy [21].

In univariate analysis, numerous clinical factors, as well as those related to irradiation, had statistically significant impact on treatment results (Tables 2 and 3). Infiltration of the anterior commissure was related to a significant decrease of LC rates and DSS rates. In case of infiltration, the risk of local relapse was 3.2 times higher than in patients without infiltration. Numerous authors have also observed that anterior commissure infiltration was correlated with a two- to three-fold increase of local relapse risk [6, 22–26]. Some authors suggest that negative impact of this factor can be diminished by delivering higher biologically effective dose (BED) and use of hypofractionation (>2 Gy) [22]. According to some researchers, infiltration of anterior commissure had no impact on treatment results [8, 19]. Mendenhall et al. concluded that infiltration of anterior commissure is usually related with larger tumor size and this parameter is responsible for worse outcomes [8].

In the analyzed material, patients over 60 years of age accounted for more than half of the group. In univariate analysis, age below 60 years had significantly negative impact on local control and disease-specific survival but significantly positive impact on overall survival (Table 2). In multivariate analysis, the impact of age and performance status on overall survival was confirmed. Local efficacy of radiotherapy in patients with T1 glottis cancer is high and such treatment is well-tolerated even in elderly patients due to the small treated volume. In elderly patients with early laryngeal cancer worse performance status is often related to comorbidities, not to cancer itself. Stokes et al. also reported a negative impact of more advanced age in patients with early glottic cancer on the risk of death. Compared to the group of patients below 60 years of age, the risk was 1.35 times higher for the group of patients aged between 61 and 70 years, and 2.29 times higher for the group of patients over 70 years [27]. In the analyzed group of 258 patients with early glottis cancer, Robert et al., reported negative impacts of age over 65 years and worse (ZUBROD 2 and 3) performance status on overall survival [28]. In their meta-analysis, Eskiizmir et al. did not show any impact of age on treatment results [26].

In our material, smoking has proven to be another factor having negative impact on treatment results. In the analyzed group, 86% of patients smoked cigarettes, and average smoking time was 34 years. Smoking was associated with an increased risk of local recurrence as well as with a reduced rate of DSS. Tobacco smoking is a basic etiologic factor of head and neck cancer. The upper surfaces of vestibular and vocal cords are most exposed to tobacco smoke, and the risk of development of larynx cancer for smokers is 7‑fold higher than for non-smokers [29, 30]. Al-Mamgani et al. showed that smoking continuation following treatment had a negative impact on local control in patients with early glottis cancer. Those patients had increased risk of developing second malignancy, and worse voice quality and overall survival [31, 32]. In their meta-analysis, Eskiizmir et al. also showed a negative impact of smoking during radiotherapy and following its completion [26].

In the analyzed group of patients, mean hemoglobin level prior to radiotherapy was 13.4 g/dl (range 9.8–16.7 g/dl). In the univariate and multivariate analysis, this factor significantly affected the chance of LC and DSS. In the previously mentioned meta-analysis, Eskiizmir et al. showed higher probability of treatment failure in patients with low hemoglobin level [26]. Other authors have also reported negative impact of low hemoglobin levels prior to therapy on treatment results in patients with early glottis cancer [31, 33, 34]. Al-Mamgani et al., in a group of patients with early glottis cancer, showed a negative impact of anemia prior to therapy on 10-year overall survival rates: 52% in the patients with anemia versus 68% in patients with a normal hemoglobin level [31]. Składowski et al. reported a 5‑year recurrence-free survival rate of 84% in the group of 235 patients with T1 glottis cancer treated with irradiation. Decrease in hemoglobin level by 1 g/dl (from 13.8 to 12.8 g/dl) reduced the probability of cure by 6%, provided that overall treatment time was 45 days [34]. Decrease of hemoglobin level, resulting in hypoxia within the tumor, has a negative impact on radiotherapy efficacy. Hall and Giaccia claim that application of at least three times higher dose of irradiation is necessary to achieve the same effect in hypoxic cells, compared to oxygenated cells [35].

In the analyzed group, malnutrition (defined as BMI <18.5 kg/m^2^) prior to therapy had a significant negative impact on 5‑ and 10-year local control, disease-specific survival and overall survival rates, compared to the groups of patients with normal nutritional status and overweight patients. In patients with head and neck cancer, malnutrition is frequent, resulting in worse outcomes and poorer quality of life. Weight loss prior to therapy is often related to more advanced disease at diagnosis and worse prognosis [36–38]. Hollander et al., in a systematic analysis of 8306 patients with head and neck cancer, showed that elevated BMI was associated with higher overall survival rates and lower risk of disease-related death and recurrence compared to underweight patients and those with normal body weight [37]. Zhao-Qu Li et al., in a group of 473 patients with laryngeal cancer, showed that BMI had significant impact on prognosis: 5‑year OS rates for groups with overweight, normal body weight and malnutrition were 87.2, 78, and 34.9%, respectively [38].

Fraction size had significant impact on treatment results (Table 2). For normofractionated radiotherapy (≤2 Gy), the risk of local failure was 3.0 times higher and the risk of cancer-related death was 3.1 times higher. Numerous randomized trials performed in patients with early glottic cancer showed that higher fraction doses can lead to improved treatment results [39–42]. Short et al. observed a 20% increase in the 5‑year LC in patients with non-advanced glottic cancer who received hypofractionated radiotherapy [40]. In a prospective trial, Yamazaki et al. achieved a 5‑year local control of 92% for fraction dose 2.25 Gy, compared to 77% for dose 2 Gy. The authors did not show any difference in 5‑year cancer-specific survival rates (100 and 98%, respectively) and 5‑year overall survival (88 and 87%, respectively). Toxicity was similar for both fraction doses. The authors stated that a higher fraction dose, with a shorter total treatment time, provides better local control [39]. Trevor et al. in the group of 10,212 patients with T1 glottic cancer noted a 2.2% improvement in 5‑year OS after hypofractionated radiotherapy [41]. Ermis et al. calculated the biologically effective dose value including treatment duration (BED) for the various fractionation schedules used in the treatment of T1 glottic cancer, showing that the BED value is similar for the tumor. However, the calculated BED for tissues reacting with late radiation reaction (α/β 3) for total dose of 55 Gy in 20 fractions was 105.4 Gy, while for a total dose of 70 Gy in 35 fractions it was 116.6 Gy, which may explain the obtained therapeutic benefit in case of hypofractionation [42]. Different biologically equivalent doses can be determined for different dose fractionation schedules from a linear-square model. The α/β index describes the individual sensitivity of various cancers and healthy tissues to a change in the df value. The value of df and the frequency of its repetition determines the difference in the response of tumor and healthy tissues to irradiation. For head and neck cancer, the value of α/β ≥ 10 Gy is assumed. Late reacting tissues (α/β = 3 Gy) are much more sensitive to even minor changes in df and its repetition more often than once a day. In patients with early glottic cancer, higher fraction doses are well-tolerated because of small volume of healthy tissues in irradiated field [35, 43]. Application of higher fraction doses shortens overall treatment time (OTT), which leads to reduction of repopulation and, as a consequence, to better treatment outcomes. In the hypofractionation schedules used in non-advanced glottic cancer OTT is shorter than in conventional radiotherapy and is 3.5–5 weeks on average, so the effect of accelerated repopulation that begins at about 4 week of radiotherapy does not play a significant role here. In head and neck cancer, it was demonstrated that at the end of the 5–7 week of conventional radiotherapy, the significant lethal effect of some given doses is compensated by the intensive repopulation of surviving clonogenic cells. The shortening of OTT in the case of hypofractionated radiotherapy may be responsible for improving the results of treatment rather than fraction dose [44].

Another negative prognostic factor in our study was the time between collecting tumor specimen for histopathology and beginning of radiotherapy. The mean time was 56 days (range 16–145 days). Prolonged waiting time for treatment may lead to tumor growth and, as a consequence, to decreased chance of cure. This issue was analyzed in numerous trials. Some authors did not observe any influence of treatment delay shorter than 44 days on results [45–47]. Others suggested that delay exceeding 30 days may lead to poorer treatment results [48–51]. In a meta-analysis of 46 studies (16 thousand patients), including 12 trials concerning head and neck cancer, Huang et al. showed that delay of radiotherapy leads to higher rates of local relapse [52]. Chen et al. performed a meta-analysis assessing impact of waiting time for therapy on outcomes in different types of cancer. For head and neck cancer, the risk of relapse rose by 0.15 with each month of waiting time for treatment [53]. Comparison of treatment results in groups of patients starting radiotherapy with different delay may only be retrospective, which weakens the value of the results obtained and the conclusions made. The influence of prolonged waiting time for radiotherapy on treatment results in patients with non-advanced glottic cancer may also indicate that the dynamics of the development of glottic cancer is not as slow as previously suggested.

## Conclusions

Radiotherapy is highly effective treatment method in patients with T1N0M0 glottic cancer. LC and DSS may be improved following hypofractionation, smoking cessation, and shortening of waiting-time until start of treatment. OS was mainly influenced by nutritional and performance status.
